# IMSA: Integrated Metagenomic Sequence Analysis for Identification of Exogenous Reads in a Host Genomic Background

**DOI:** 10.1371/journal.pone.0064546

**Published:** 2013-05-23

**Authors:** Michelle T. Dimon, Henry M. Wood, Pamela H. Rabbitts, Sarah T. Arron

**Affiliations:** 1 Department of Dermatology, University of California San Francisco, San Francisco, California, United States of America; 2 Leeds Institute of Molecular Medicine, St James’s University Hospital, Leeds, United Kingdom; Auburn University, United States of America

## Abstract

Metagenomics, the study of microbial genomes within diverse environments, is a rapidly developing field. The identification of microbial sequences within a host organism enables the study of human intestinal, respiratory, and skin microbiota, and has allowed the identification of novel viruses in diseases such as Merkel cell carcinoma. There are few publicly available tools for metagenomic high throughput sequence analysis. We present Integrated Metagenomic Sequence Analysis (IMSA), a flexible, fast, and robust computational analysis pipeline that is available for public use. IMSA takes input sequence from high throughput datasets and uses a user-defined host database to filter out host sequence. IMSA then aligns the filtered reads to a user-defined universal database to characterize exogenous reads within the host background. IMSA assigns a score to each node of the taxonomy based on read frequency, and can output this as a taxonomy report suitable for cluster analysis or as a taxonomy map (TaxMap). IMSA also outputs the specific sequence reads assigned to a taxon of interest for downstream analysis. We demonstrate the use of IMSA to detect pathogens and normal flora within sequence data from a primary human cervical cancer carrying HPV16, a primary human cutaneous squamous cell carcinoma carrying HPV 16, the CaSki cell line carrying HPV16, and the HeLa cell line carrying HPV18.

## Introduction

Metagenomics, the study of microbial genomes within diverse environmental samples, has rapidly developed as a field since its introduction in 1998[1]. In 2012, a keyword search on the term in Pubmed yielded over 1,200 articles, with topics ranging from large environmental surveys to focused medical samples. Rapid advances in high throughput sequencing have enabled acquisition of large genomic datasets at reasonable cost, allowing explosive advances in sequence-driven metagenomic research. Secondary analysis of publicly available sequence datasets is also increasing as analysis tools become available, and software for analysis of metagenomic sequence datasets has had to keep pace with these rapid developments.

A key area of metagenomics is the identification of microbial sequences within a larger host organism. These studies have enabled the study of normal and diseased human intestinal, respiratory, skin and urogenital microbiota[2], [3], [4], identification of novel viruses in diseases such as human Merkel cell carcinoma and acute hemorrhagic fever[5], [6] and in a variety of animal diseases including avian proventricular dilatation disease, snake inclusion body disease, and bee colony collapse[7], [8], [9]. A crucial element of the analysis of metagenomic sequence data derived from a host organism is the detection of non-host sequences within a complex host genomic background. These exogenous sequences may represent potential pathogens, commensal organisms, or laboratory contaminants such as vector sequence.

Large sequencing laboratories frequently develop an analysis pipeline specific to the needs of the project at hand, often requiring computing power in excess of what individual laboratories can support. A number of groups describe general analysis methods in which host reads are “subtracted” from the sequence readset by homology to the human genome. These methods typically use public tools such as BLAST or Bowtie[10], [11] in combination with proprietary code written by the authors[7], [12], [13], [14]. There are few tools available to groups with less experience in software development for high throughput sequence analysis. PARSES (Pipeline for Analysis of RNA-Seq Exogenous Sequences) is a system that uses BLAST+ for rapid filtering of human reads followed by MEGAN for visualization of metagenomic data[15]. PARSES is designed to work on a 64-bit desktop computer, though with limited memory the time required for analysis of a single dataset can require multiple days. It requires Novoalign, a paid-license software, for alignment. PathSeq, a computational subtraction method offered by the Broad Institute, relies on the Amazon cloud computing environment to expand the computational power, but there are significant associated costs[16].

Other available tools are limited to analysis of host-filtered data. MGAviewer is tool for metagenomic alignments, which can be used for visualization of alignment data[17]. This tool is web-based, requiring no software installation; however it requires that the user have the expertise and computational equipment to produce the host filtered alignment data to be visualized. MetaSAMS is an extension of SAMS (Sequence Analysis and Management System), a system that aggregates other tools for individual sequence reads or used-assembled contigs. MetaSAMS also requires user host filtering prior to use[18].

The optimal system for metagenomic sequence analysis would isolate exogenous sequences from a complex host genomic background and characterize those sequences by taxonomic classification. To apply to multiple study designs from different research fields, the system would need to have flexibility in user-selected and updatable databases, levels of stringency in mapping, and a variety of filtering options. It would be fast and comprehensive, with intelligible output, post-processing functionality, and would be scalable to laboratories running analysis on computer clusters as well as those without.

This paper describes Integrated Metagenomic Sequence Analysis (IMSA), a computational analysis pipeline that meets the above criteria and is available for public use (SourceForge). IMSA takes input sequence from high throughput datasets and utilizes a user-defined host database to filter out host sequence. IMSA then aligns the filtered reads to a user-defined universal database to characterize exogenous reads within the host background. IMSA assigns a score to each node of the taxonomy based on read frequency, and can output this as a taxonomy report suitable for cluster analysis or as a taxonomy map (TaxMap). IMSA can also output the specific sequence reads assigned to a taxon of interest for downstream analysis.

## Algorithm

### IMSA uses an Action File to filter non-host reads and align to universal database

IMSA uses an action file to guide the filtering and alignment steps. A typical action file to filter a large read set against the human genome might be:

quality

bowtie human doDivide = True

blat human | -fastMap

blat human

blast human maxEval-1e-15 | -word_size 24

blast human maxEval-1e-8

blast nt maxEval-1e-5 | —max_target_seqs 200

For each step there are additional parameter options which are described in the user manual.

Quality filtering by default removes all reads with more than 15 bases with a quality less than 15. The user can define alternate quality metrics or omit this step from the action file. For example, “quality 10 20” would remove reads where more than 10 bases had a quality score less than 20.

For alignment actions, each line in the action file specifies two steps. First, alignment is performed with the specified alignment program, such as bowtie or BLAST. Next, the read set is filtered to remove reads with a hit in the alignment results. Parameters for filtering are before the pipe (“|”) whereas anything after the pipe is sent directly to the alignment program. Some parameters, such as “maxEval” for blast can be used for both filtering and alignment and will automatically be sent to the alignment program. By default paired ends are treated as individual reads for maximum sensitivity, but this behavior can be modified. Databases for the alignment programs are defined in the IMSA configuration file so the databases used and their location in the computer system can be easily modified. Similarly, the configuration file can specify an ooc file for use in the blat alignment.

IMSA can run and filter bowtie, blat and blastn (new NCBI version) alignments. Filtering the read set using the bowtie results can be memory intensive so an option to divide the file into pieces can be used to reduce the memory footprint. Blat and Blast alignments can be performed on a SUN Grid cluster or run straight, without a cluster.

The final step in the action file is the alignment to the universal database. The input to this alignment is the host-filtered read set. For this step, the blast results are used in subsequent analysis, rather than simply being used to filter the read set.

For host filtering blast steps, the default is to set the max_target_seqs parameters to 5 to short circuit searches for reads that map multiple places. In the alignment to the universal database, seen above on the last line of the action file, this parameter is set higher so all matches for each read are found.

### Taxonomy scores are calculated based on universal database alignment and visualized with TaxMaps

Once IMSA has completed the filtering action file, the next step is to process the universal database alignment results to yield the IMSA reports. First, the blast results are processed to identify the best alignment for each read. If a read hits many sequences in the universal database, only the alignment with the highest score is kept. If a read aligns to multiple sequences with equally high scores, all of the targets are reported. However, IMSA assigns a score to each target indicating whether and how much the sequence read is split. For example, a read that aligns to a single sequence in the universal database is given a score of 1.0. If a read hits two sequences in the universal database with equal scores, both alignments get a score of 0.5. A read hitting three sequences will get a score of 0.333 for each hit, etc. This treatment allows ties to be kept, but the score assigned to each node is lower for non-unique reads that likely represent conserved regions, and higher for reads that are unique to that taxonomic node. The default behavior is to keep all the ties scored in this way, but IMSA provides the functionality to filter reads with scores below a given threshold. Though this reduces sensitivity, it can be informative to only look at reads with a unique best hit in the universal database.

Next, the taxonomy of the scored blast result file is calculated. For this, the species of each hit in the universal database is determined from the gi. For this step, IMSA assumes that the universal database is the NCBI nucleotide transcript database (nt)[19]. If the universal database is another database with titles in a different format, the user will can customize the portion of the code that extracts a gi from the fasta title and translates it into a species. Once the targets are determined for each hit, IMSA then retrieves the entire taxonomic record for those targets from NCBI. IMSA uses the best read alignment scores assigned to each target to calculate the score for each species, genus, family and division with an aligned read in the sequence dataset. The taxonomy of universal database results is listed in taxonomy report text files. In addition, TaxMap bubble diagrams can be generated for species, genus, family and division. IMSA generates text files in a format ready to be interpreted into a diagram using the GraphViz open source graphing software. These data can be used to for downstream analysis to characterize the metagenome of the sample or to identify potential pathogens.

IMSA includes additional tools for subsequent investigation. The python script getFastaForTaxonomy takes a list of taxonomy IDs (at any level) along with the fasta file of filtered reads and the blast alignment to the universal database to create a fasta file of all the reads aligning to the taxonomic IDs. The script speciesToClusterTable can take a set of species, genus, family, or division files and create a table of results suitable for input into Cluster and TreeView for visualization of the frequency in each sample of a larger study[20], [21].

## Results

IMSA was used to analyze sequence data from four previously published datasets to demonstrate the ability to detect human papillomavirus in a variety of settings (Table 1). Genomic DNA from the CaSki cell line, which contains HPV16, was sequenced with 70 bp single end reads[22]. RNA-seq data was analyzed from 150bp paired-end reads from three samples: the HeLa cell line, which contains HPV18, a primary cervical cancer containing HPV16, and a primary periungual squamous cell carcinoma containing HPV16[23].

**Table 1:** Overview of IMSA results on positive control datasets.

Dataset	Source	Expected Virus	Total Reads	% Host filtered	% Total Viral reads	Viral Reads in filtered set	% Total Viral Reads Detected by IMSA
CaSki Cell Line	Genomic DNA, Single end	HPV16	1.9E+06	99.4	0.0014	2447	91
HeLa Cell Line	RNA-seq, Paired end	HPV18	9.2E+05	98.3	0.0032	2755	94
Primary Cervical Carcinoma	RNA-seq, Paired end	HPV16	2.0E+06	97.8	0.0004	631	86
Primary Cutaneous Periungual SCC	RNA-seq, Paired end	HPV16	7.8E+05	96.3	0.0012	924	98

The action file specified for these examples consisted of a quality filtering step followed by Bowtie, two iterations of Blat with increasing stringency, four iterations of BLAST, and an alignment to nt as the universal database, as shown below:

quality

bowtie human

blat human | -fastMap

blat human

blast human maxEval = 1e-18 | -word_size 40

blast human maxEval = 1e-15 | -word_size 32

blast human maxEval = 1e-10 | -word_size 24

blast human maxEval = 1e-8

blast nt maxEval = 1e-15 | -max_target_seqs 200

**Figure 1:**
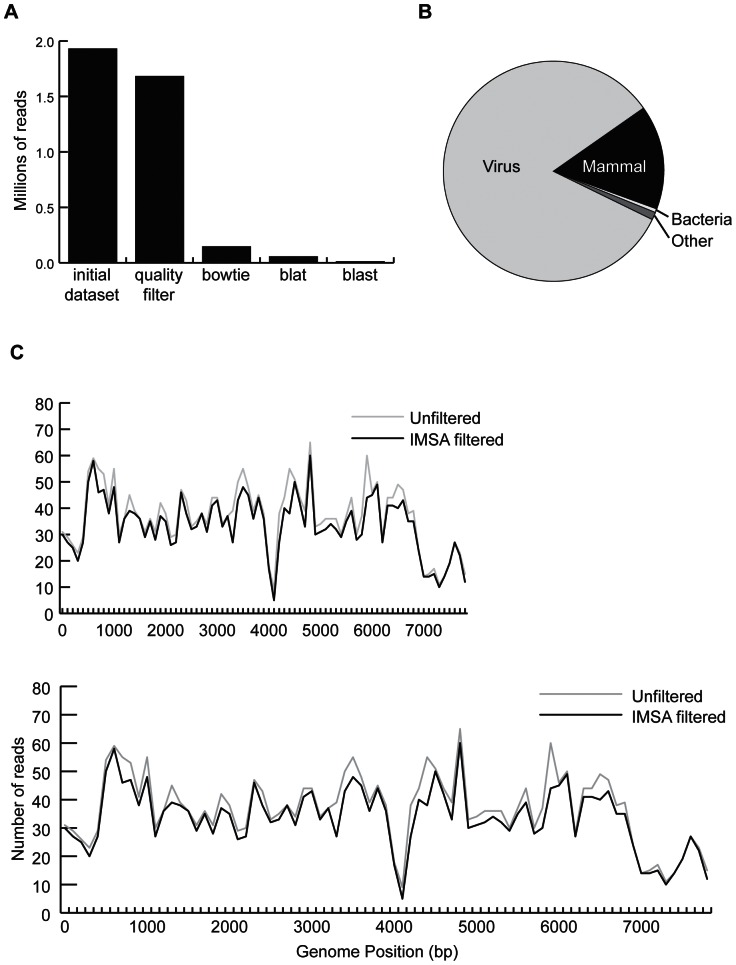
IMSA results on CaSki positive control dataset. A) Bar chart showing the number of reads in the dataset at each step of the IMSA pipeline. B) Breakdown of the division of reads left after host filtering, as determined by BLAST to NCBI’s nt database. C) The number of reads that align within each 100 base pair bin along the HPV16 genome in the unfiltered dataset compared to the IMSA filtered dataset.

Output for the CaSki cell line is shown in Figure 1. After host filtering (Figure 1a), the remaining reads were mapped to nt (Figure 1b). The majority of non-host reads were viral; the fraction of mammalian reads left over after host filtering represented sequence that was likely human derived but differed due to low quality or sufficient divergence from human to pass host filtering steps. IMSA captured 91% and 94% of the HPV reads present in the cell line datasets and 86% and 98% of the HPV reads present in the primary cancer datasets. Viral reads that were removed in host filtering were primarily those removed for poor quality; Figure 1c demonstrates unbiased loss of viral reads across the HPV genome in quality filtering.

**Figure 2:**
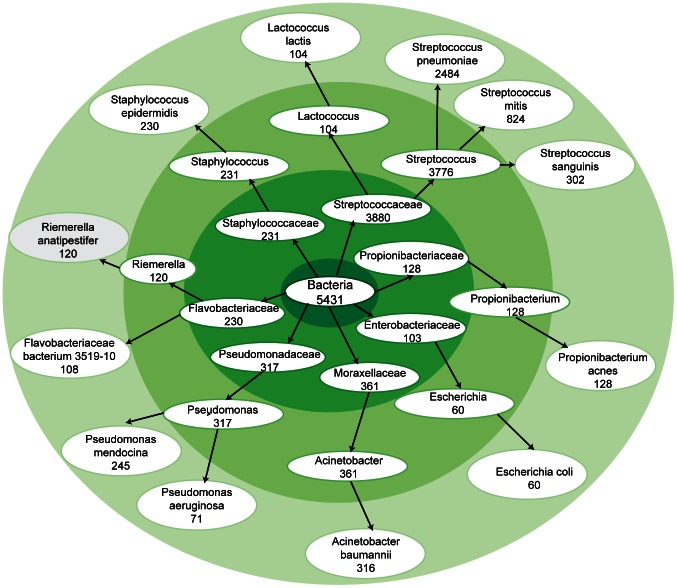
TaxMap of bacterial reads in a primary cutaneous SCC. TaxMap of shows the breakdown of bacterial read scores at the kingdom, family, genus and species levels. This TaxMap has been filtered to only show nodes with a score above 50.

Our method for quantifying exogenous sequence goes beyond a simple read count to create a score for each node of taxonomy (Figure 2). This method allows reads in conserved regions of pathogen genomes to contribute fractional scores to multiple species; a score of 1 is assigned only when the read is a unique match to a single species. Additional options allow the user to define the maximum number of mapped ties allowed. Allowing ties is optimal for exploratory analysis; evaluating uniquely mapped reads will increase specificity in scoring but reduce sensitivity.

**Figure 3:**
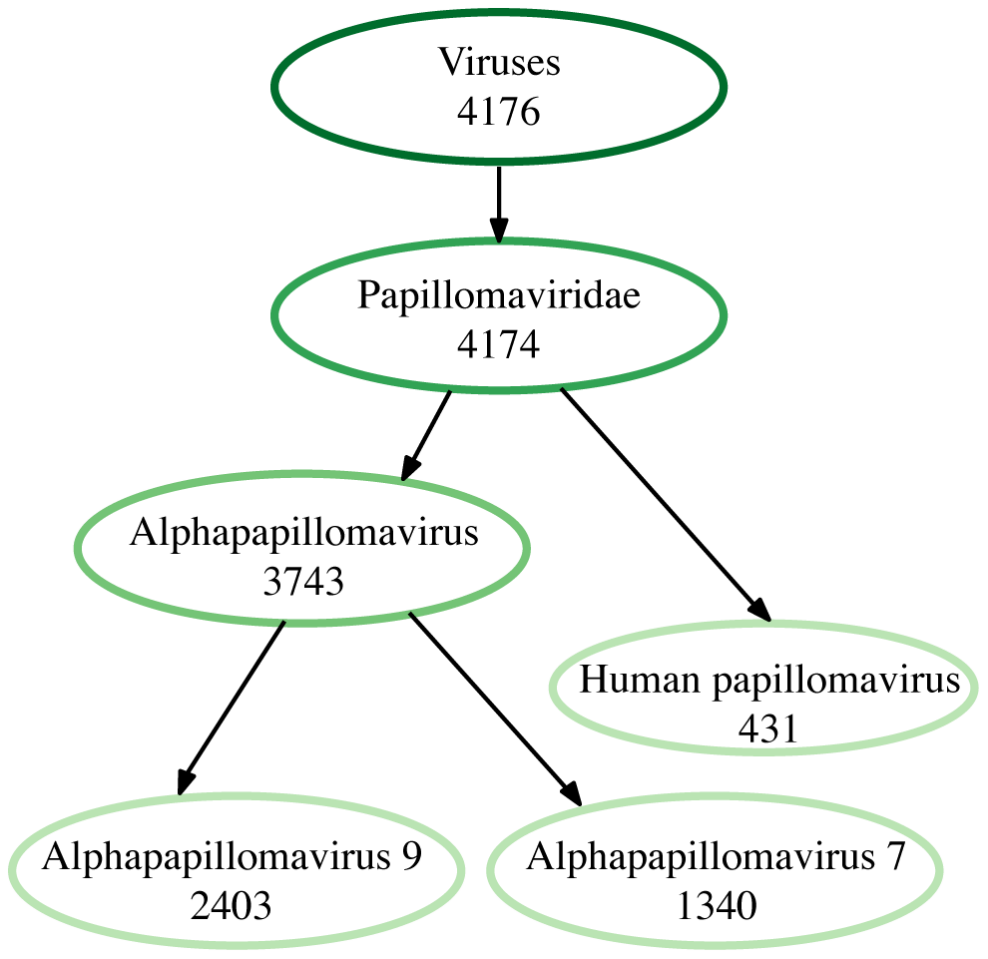
TaxMap of viral reads in a combined HeLa and CaSki dataset. IMSA is able to accurately identify both alphapapillomaviridae species 7 (HPV18) and species 9 (HPV16) in the merged dataset.This TaxMap has been filtered to only show nodes with a score above 50.

To determine the accuracy for a read library containing multiple strains of the same organism, we combined the data from the two readsets derived from HeLa and Caski cells. Because these libraries were prepared and sequenced differently, we pulled the forward read of the 54bp, paired end HeLa library and the first 54 bases of reads in the 70bp single end CaSki library into a single dataset for analysis. IMSA accurately identified both alphapapillomaviridae species 7 (HPV18) and species 9 (HPV16) in the merged dataset at the expected frequency (Figure 3).

**Figure 4:**
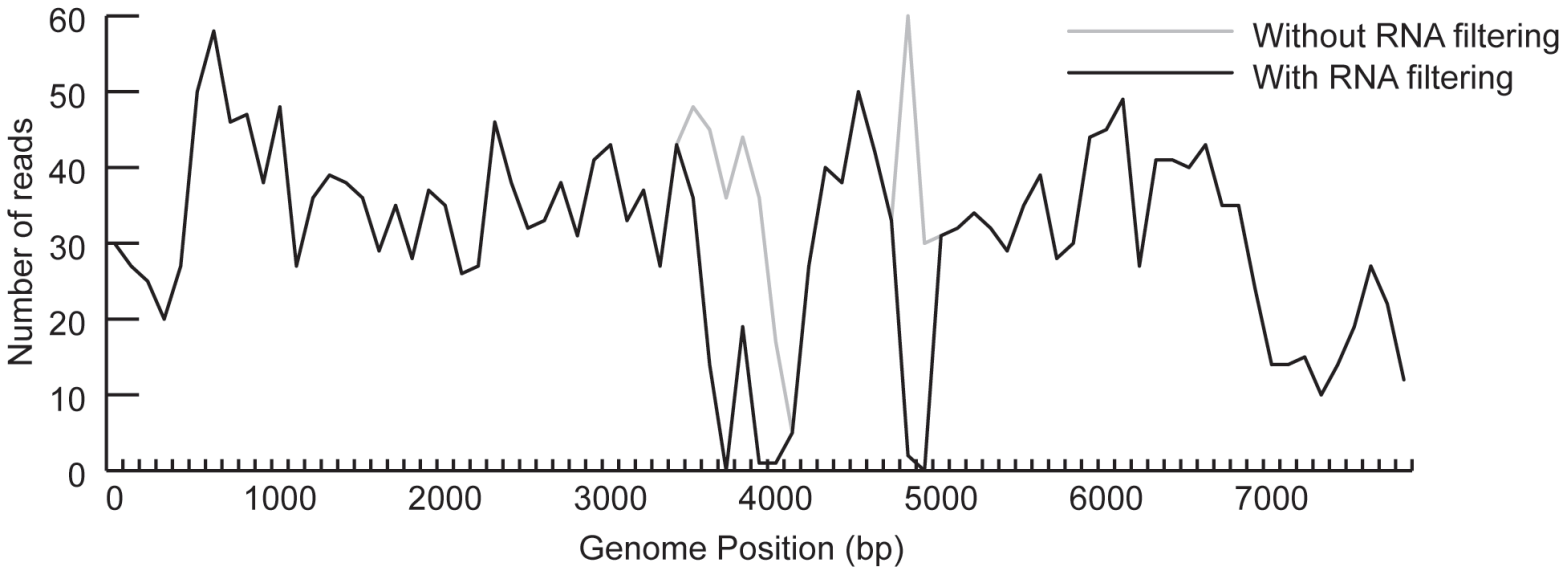
Comparison of filtering databases. NCBI’s RefSeq database includes viral sequence mis-annotated as human; using this as a host filter results in loss of HPV16 reads (black). Filtering against the human genome (hg19) alone allows detection of these reads (gray).

The choice of host filtering database(s) will impact the results obtained. Figure 4 demonstrates CaSki cell line data filtered against the human RefSeq RNA database as well as HG19, to remove reads annotated as human. However, at three positions on the viral genome the number of viral reads detected drops to zero. A parallel attempt to filter against a HG19 alone reveals HPV16 reads in those regions. This is due to misannotation in the RefSeq human RNA database, in which sequences are annotated as human but contains HPV16 sequence (in specific, we found gis 12300658, 12301139, and 12306164 to contain HPV16 sequence annotated as human but this is not an exhaustive list). This misannotation results in viral reads being removed as “host” when filtered against a database of human RefSeq sequences.

## Discussion

We present IMSA, a system for Integrated Metagenomic Sequence Analysis of high throughput sequence data. IMSA can be used to analyze data from genomic or RNA-seq datasets. IMSA takes a user-defined action file to isolate exogenous sequences from a host genomic background. These sequences are characterized by taxonomic classification and can be delivered in a taxonomy report or visualized with TaxMap.

The taxonomy report delivers a comprehensive picture of the exogenous reads present in each sequence dataset. The use of a universal database for characterizing non-host reads is improvement over metagenomic analysis techniques that screen sequence data solely against curated bacterial or viral databases. TaxMap provides a visual method for evaluating the diversity of exogenous species present.

Our post-processing functionality includes the ability to retrieve reads derived from a taxon of interest with the python script getFastaForTaxonomy. If the results reveal a number of reads aligning to a particular species of interest, obtaining the fasta file of all reads from that species allows ready assembly with tools such as Edena[24], or design of PCR primers for validation studies. In addition to identifying and quantifying the relative abundance of pathogen sequence, the isolated reads aligning to the target pathogen can be used for more detailed downstream analysis. For example, reads derived from RNA-seq data could be used to determine relative pathogen gene expression levels or to identify common gene pathways expressed by pathogen in a sample set. This is a benefit of IMSA over 16S-based pyrosequencing.

The python script speciesToClusterTable will output IMSA scores in a format for Cluster /Treeview analysis. Looking at the species results across multiple samples can frequently yield clear patterns that may not be visible looking at individual results. For example, a particular species may not be the most common result, or may be found across all the samples, but examination with Cluster/TreeView may show that the species is consistently more common in samples in a specific state (i.e. all the diseased samples). Filtered IMSA files can also be used with other existing tools such as MGAviewer, metaSAMS, and MEGAN.

IMSA is scalable to small or large laboratories. It can be run on a desktop computer or on a SUN Grid cluster. The time required to process a sample is dependent on multiple parameters including the size of the dataset, the filtering parameters, and the computational power allocated to processing. Certain steps such as the Bowtie and BLAST can be run in parallel on multiple processors, while others such as Blat are single threaded. In addition, certain steps are fixed, such as loading the human genome into memory, while others will scale with readset size. Thus the speed of processing does not necessarily scale in a linear fashion with dataset size- smaller readsets will take more time per million reads but less time overall.

We calculated processing speeds based on various ongoing analyses in our laboratory. 50bp single end read data required 4:38 hours per million reads per node; 30 million reads run on 20 processors took 6:57 hours on the wall clock. 80bp single end read data required 6:32 hours per million reads per node; 30 million reads run on 5 processors took 39:34 hours on the wall clock. 50bp paired end read data required 1∶58 hours per million reads per node; 150 million reads run on 10 processors took 19∶31 hours on the wall clock. In this study, the analysis described above was done on a Linux cluster with SUN Grid Engine. The CaSki dataset included 2 million single end reads and took 57 minutes to analyze using 5 processors. The primary cervical carcinoma dataset included 2 million paired end reads and took 47 minutes to analyze using 10 processors.

The action file is designed to be flexible, allowing the user to select the appropriate host database for filtering and universal database for mapping exogenous reads. With the filtering parameters described in our test case, an 86% detection efficiency would translate to a probability of failing to detect any reads from a virus present in 10 reads at 0.14^10^, or 1 in 3×10^9^. Given IMSA’s flexible algorithm, the sensitivity can be increased by reducing the stringency of the filter. Decreased stringency speeds host filtering and allows more potential pathogen reads, but delays downstream analysis as host reads that pass filters will slow mapping to the universal database. In contrast, increased stringency in host filtering requires more time and computational resources, but thoroughly removes host reads before potential pathogen reads are mapped to the universal database. The tradeoff is that potential pathogen reads may be lost in stringent filters. Our laboratory typically optimizes the action file for each experiment by splitting out 10% of the sequence dataset and running test action files to quickly assess the efficiency of filtering with a variety of parameters before moving forward with the complete readset. Our experience is that the most efficient method combines the initial speed of low stringency filtering steps on the total dataset followed by successively more stringent filters as the dataset becomes smaller with each step. IMSA is designed for use on Illumina datasets, but is flexible enough to be used on 454 read sets by adjusting the action file alignments and parameters for the longer 454 read length.

Overall, IMSA delivers a flexible, comprehensive method for metagenomic analysis of high-throughput sequence datasets. It is a valuable addition to existing tools for the rapidly growing field of metagenomic research.
